# Materia Medica and Therapeutics; Medical Chemistry; Toxicology and Legal Medicine

**Published:** 1860-03

**Authors:** Emil Fischer


### MATERIA MEDICA AND THERAPEUTICS; MEDICAL CHEMISTRY; TOXICOLOGY AND LEGAL MEDICINE.

BY EMIL FISCHER, M.D.

### Materia Medica and Therapeutics.

I.—*On the Dietetic and Medicinal Properties of Erythroxylon Coca.* By Dr. Mantegazza. (Prize Essay. Pamphlet. Milan, 1859.)

The erythroxylon coca, a plant which grows in moist and woody regions on the eastern slopes of the Andes, is highly valued by the inhabitants of Peru, Chili, and Bolivia, not only as a medicine, but also as an article of food; and serves with them as a substitute for the tea, coffee, betel, tobacco, hashish, and opium, used by other nations. Its culture, upon which, since the time of Pizarro's conquest, much care has been bestowed, has recently increased to such a degree, that in the year 1856 the revenue of the Republic of Bolivia, from the sale of this herb, amounted to thirteen millions of francs—a very large sum, if compared with the small number of consumers, (800,000.) According to the account of M. Pöppig and of other well-known travelers, the natives use the dried leaves of the coca-plant either by themselves or in combination with a highly-alkaline substance called llicta, which is prepared from roasted potatoes and the ashes of different other plants; they masticate them like the Malays and the inhabitants of the Indian Archipelago do the calcined leaves of the piper betel. The use of this masticatory, which is considered a great delicacy, is not, however, confined to the rich; on the contrary, it is particularly among the hard-working Indians that the coca enjoys a high reputation as a nutriment and restorative, and its use is considered absolutely essential for the endurance of fatigue and exertion, so that a laborer in making his contract has a view not only to wages, but to the amount of coca to be furnished. The Inca, who lives at a height of seven to fifteen thousand feet above the level of the sea, and whose meagre fare consists principally of maize, some dried meat, and potatoes of bad quality, believes that he can sustain his strength solely by the use of coca; the porter who carries the mail, and accompanies the traveler over the roughest roads at the quick pace of the mule, invigorates and strengthens himself by chewing coca; the Indian who works half naked in the silver and quicksilver mines, looks upon this plant as an ambrosia capable of imparting new life, and of stimulating to new exertions. It is not surprising, under such circumstances, that this article should be very much abused, and that the evil of intemperance in the use of coca, known as coquear, should be quite as prevailing among the natives of those districts as intemperance in the use of tobacco, alcoholic liquors, and opium is among other nations. They often intoxicate themselves for several weeks, hide in the deepest forests in order not to be disturbed in their enjoyment, and not rarely return home to their family suffering from delirium, or decided idiocy.

The child and the feeble old man seize with equal eagerness the leaves of the wonderful herb, and find in it indemnification for all suffering and misery. Be it that the praised efficacy of the plant is merely the effect of fancy or tradition, or that the plant really contains a powerful principle unknown to science, the solution of this mystery is certainly a worthy theme for scientific inquiry, and the investigations of Dr. Mantegazza deserve, therefore, our full attention.

Dr. Mantegazza observed that the chewing of a drachm of the leaves of the coca increased salivation, giving at first a somewhat bitter, and afterward an aromatic taste in the mouth, and a feeling of comfort in the stomach, as after a frugal meal eaten with good appetite. After a second and third dose, a slight burning sensation in the mouth and pharynx, and an increase of thirst were noticed; digestion seemed to be more rapidly performed, and the feces lost their stercoraceous smell, the peculiar odor of the juice of the coca becoming perceptible in them. On using the coca for several days, the author observed on himself as well as on other individuals a circumscribed erythema, an eruption around the eyelids resembling pityriasis; from time to time a not unpleasant pricking and itching of the skin was felt. An infusion of the leaves, taken internally, was found to increase the frequency of the pulse in a considerable degree. In making observations on the frequency of the pulse, the author was very careful to consider all the conditions which might influence it; he found that the temperature of the air being the same, and the liquids being heated to an equal degree, an infusion of coca will increase the action of the heart four times its normal standard, while cocoa, tea, coffee, and warm water only double it. By taking an infusion prepared from three drachms of the leaves, a feverish condition was produced, with increased heat of the skin, palpitation of the heart, seeing of flashes, headache, and vertigo; the pulse rose from seventy to one hundred and thirty-four. A peculiar roaring noise in the ear, a desire to run about at large, and an apparent enlargement of the intellectual horizon indicated that the specific influence upon the brain had commenced. A peculiar, hardly describable feeling of increased strength, agility, and impulse to exertion follows; it is the first symptom of the intoxication, which is, however, quite different from the exaltation produced by alcoholics. While the latter manifests itself by increased but irregular action of the muscles, the individual intoxicated by coca feels but a gradually augmented vigor, and a desire to spend this newly-acquired strength in active labor. After some time the intellectual sphere participates in this general exaltation, while the sensibility seems to be hardly influenced; the effect is thus quite different from that produced by coffee, and resembles in some degree that of opium. Dr. Mantegazza could, in this excited condition, write with ease and regularity. After he had taken four drachms he was seized with the peculiar feeling of being isolated from the external world, and with an irresistible inclination to gymnastic exercise, so that he who in his normal condition carefully avoided the latter, jumped with ease upon the writing-table without breaking the lamp or other objects upon it. After this a state of torpidity came on, accompanied by a feeling of intense comfort—consciousness being all the time perfectly clear—and by an instinctive wish not to move a limb during the whole day, not even a finger. During this sensation sleep sets in attended by odd and rapidly changing dreams; it may last a whole day without leaving a feeling of debility or indisposition of any kind. The author increased the dose to eighteen drachms in one day; his pulse rose in consequence of it to one hundred and thirty-four, and in the moment when the delirium was most intense, he described his feelings to several of his colleagues, who observed him, in the following written words; "*Iddio é ingiusto perche ho fatto l'uomo incapace di poter vivere sempre cocheando*," (this is the expression for intoxication by coca.) "*Io preferisco una vita di* 10 *anni con coca che un di* 1,000,000 *Secoli senza coca*." After three hours of sleep Dr. Mantegazza recovered completely from this intoxication, and could immediately follow his daily occupation without the least indisposition—on the contrary, even with unusual facility. He had abstained for forty hours from food of any kind, and the meals then taken were very well digested. From this fact the author finds it explainable, that the Indians employed as carriers of the mail are able to do without food for three to four days, provided they are sufficiently supplied with coca.

From these experiments made repeatedly on himself, and on other individuals, Dr. Mantegazza draws the following conclusions:—

1. The leaves of the coca, chewed or taken in a weak infusion, have a stimulating effect upon the nerves of the stomach, and thereby facilitate digestion very much. 2. In a large dose coca increases the animal heat and augments the frequency of the pulse, and consequently of respiration. 3. In a medium dose, three to four drachms, it excites the nervous system in such a manner that the movements of the muscles are made with greater ease—then it produces a calming effect. 4. Used in a large dose it causes delirium, hallucinations, and finally congestion of the brain.

The most prominent property of coca, which is hardly to be found in any other remedy, consists in the exalting effect it produces, calling out the power of the organism without leaving afterwards any sign of debility. The coca is in this respect one of the most powerful nervines and analeptics. These experiments, as well as the circumstance that the natives have used the coca, from the earliest period, as a remedy in dyspepsia, flatulency, and colic, have induced Dr. Mantegazza, and several of his colleagues in South America and Europe, to employ the leaves of the coca in a variety of cases, partly as masticatory, partly in powder, as infusion, as alcoholico-aqueous extract in the dose of ten to fifteen grains in pills, and as clyster. Dr. Mantegazza has used coca with most excellent results in dyspepsia, gastralgia and enteralgia; he employed it not less frequently in cases of great debility following typhus fever, scurvy, anæmic conditions, etc., and in hysteria and hypochondriasis, even if the latter had increased to weariness of life. The coca might also be employed with great benefit in mental diseases where some physicians prescribe opium. Of its sedative effect in spinal irritation, idiopathic convulsions, nervous erethism, the author has fully convinced himself. He proposes its use in the highest dose in cases of hydrophobia and tetanus. It is a popular opinion that the coca is a reliable aphrodisiac; the author has, however, observed only two cases in which a decided influence upon the sexual system was perceived.

Dr. Mantegazza, finally, recommends this remarkable plant, which could be easily introduced into trade, to the profession for further physiological and therapeutical experiments, and adds the full history of eighteen cases by which the medicinal virtues of the remedy are proved to satisfaction.—(*Oesterreichische Zeitschrift für Praktische Heilkunde*, November 4, 1859.)

II.—*Injection of Sulphate of Atropine on the Pneumogastric Nerve in Asthma.* By PROFESSOR COURTY.

Professor Courty, of Montpelier, has communicated to the Academy of Sciences of Paris a case wherein he used this novel kind of treatment. The patient was a lady aged fifty-four, who for several years had suffered from very severe fits of asthma. No organic disease of the heart was discovered. Relief was obtained in several fits, which occurred at three and four months interval, by emetics, purgatives, frictions with mercurial and belladonna ointments, opium, valerian, and blisters dressed with morphine, sulphurous waters, etc.

In August of this year, the fit having recurred, M. Courty injected on the internal side of the sterno-mastoid muscle, and on a level with the thyroid body, six drops of a solution of sulphate of atropine (one grain of the salt to one hundred of water,) just on the tract of the sheath which contains the vessels and the pneumogastric nerve. The trocar was introduced to the depth of only three or four lines, for fear of injuring the important vessels of the region. Symptoms of narcotism were observed, but the breathing was freer. The effects of the atropine injection lasted till the next day, when a second and similar injection was made on the right side. The narcotism now persisted during three days, and was combated by purgatives, enemata, tartar emetic, etc.; and on the fourth day, a third injection of seven drops was had recourse to, the canula being introduced a little below the former puncture on the right side, to the depth of eight or nine lines, and moved about to allow the liquid to penetrate. Strong narcotism ensued, but it did not last long, and the fit of asthma was completely controlled.—(*Lancet*, December 24, 1859.)

III.—*A few particulars respecting Woorara.* By M. BONVIER.

M. Bonvier, relying upon a work published by M. Reynoso, gave, at a late meeting of the Surgical Society of Paris, the following particulars respecting the composition of woorara:—

There is true and false woorara; the two are very different, but extremely difficult to distinguish from each other. The true woorara presents, moreover, several species; the substance is therefore not always obtained of the same strength. It comes from different countries, and is extracted from one or several plants which contain one identical principle, the character of which is to cause death when injected into the blood, and to be innocuous when taken into the stomach.

There is, however, one sort of woorara which acts on the gastric mucous membrane of certain animals at given ages, which circumstance would tend to show that much difference exists in some samples of woorara.

It is well known that it is not always prepared from the same plants, nor from plants of the same nature. It has even been shown by M. Chombrook that one kind of woorara is obtained from a great number of plants, almost as great as the number of ingredients entering into diascordium or theriacum.

Among the plants used are some strychneæ, but the rest have not as yet been determined botanically. It is doubtful whether snake poison is mixed with it.

Gunelli is the first who, in 1758, insisted upon the innocuous nature of woorara when taken into the stomach. Lacondamine and Humboldt corroborated his statements. Men can eat with impunity animals killed by woorara. Death by the poison occurs generally by paralysis of the motor nerves.

It is of importance to try the woorara in various manners before administering it to the patient, and to ascertain whether it produces no poisonous effects when introduced into the stomach, and also whether it paralyzes motor nerves without affecting the nerves of sensibility. These precautions are indispensable, for there is a kind of woorara which may kill by gastric absorption.

As to the occasional inefficacy of the poison when inocculated, the experiment of M. Dequise may be mentioned. This surgeon had been given, by a traveler, a quiver full of arrows said to be poisoned by woorara. He found, however, on trying them upon a dog, that they produced no effect. If we consider the woorara as an extract, such a result need create no surprise, as extracts are very liable to change.—(*London Lancet*, New York edit., February, 1860.)

### IV.—*Curare in Tetanus.*

The successful termination of two cases of tetanus in which curare was employed by M. Vella and M. Chassaignac, has led to repeated trials and as many failures with this potent substance. We may count in all, now, nine attempts and seven failures. Great interest has been excited by the experimental employment of curare in these cases, for the disease is no less horrible than the remedy is heroic. The discussion at the Medico-Chirurgical Society threw but little light on the subject. It is one of the least promising characteristics of curare, that both in composition and effect it is uncertain and varying. Its chemical nature is not uniform; even its source is variable, and its energies are unequal in various specimens. This constitutes a great posological objection to its use in its present form. If it were possible to distill a yet more deadly poison from this most poisonous drug, and to extract from it *curarina* as its active alkaloid, a fixed intensity might be attained. The therapeutic action of curare is unreliable; it cures rarely, but it can kill promptly. It was thought not to be poisonous when taken into the stomach; subsequent experiments have proved this to be an error. Experiments upon large animals have shown that after the tetanic spasms have ceased, perhaps controlled by the action of the curare, death has followed apparently from paralysis of the organs of respiration. So that the surgeon has the satisfaction of thinking, that if he saves his patient from the disease he may kill him by the remedy. It is a remarkable fact, that wounded and enfeebled animals are only affected by doses of greater than ordinary power. All this leads us to coincide in opinion with M. Velpeau, who condemns, rather than with M. Bernard, who applauds curare. Further experiments are promised. Perhaps it may not be superfluous to suggest that they should be made exclusively in *corpore vili*. What has been done hitherto was justified by the success of Vella and Chassaignac. The subsequent failures suggest the possibility that those were recoveries not cures.—(*London Lancet*, December 10, 1859.)

### V.—*Employment of Sambucus Nigra in Dropsy.* By M. REYSSIE.

M. Reyssie, a Belgian practitioner, states that he has long employed the juice of the root of the sambucus as an excellent purgative in dropsy. The bark of the fresh root must be detached by scraping, and the juice is extracted from the scrapings by pressure. The dose is a teaspoonful for an ordinary purgative; but as it does not cause colic or any other inconvenience, the quantity may, in the case of dropsy, be increased to a tablespoonful, which will often induce from twenty to thirty stools. It is a curious fact, that the process of boiling, as in the preparation of a syrup, converts this purgative into a diuretic, which may also be of great use in dropsy.—(*Revue Médic.*, November, 1859, and *Medical Times and Gazette.*)

### VI.—*Borax in Diphtheritis.* By M. LERICHE.

M. Leriche, having derived great advantage from the employment of large doses of borax in croup and the various pultaceous affections of the buccal mucous membrane, determined upon giving it in diphtheritis, which was prevailing in his locality. He relates two cases, occurring in adults, wherein its employment was quite satisfactory, in one of which twenty-six drachms were given in four days, and in the other fifty drachms in six days.—(*Ibid.*)

### VII.—*Alum Lozenges in Affections of the Throat.* By M. ARGENTI.

M. Argenti, of Venice, proposes as a substitute for alum gargles in affections of the throat, lozenges formed of alum, sugar, and tragacanth, mixed up with diluted laurel-water so as to form lozenges, each containing a suitable dose of alum. The mass is to be well manipulated, and after division, to be put on a sheet of paper and dried by a gentle heat. The lozenges keep well and form an agreeable medicament, which, by aid of the saliva, becomes effectually applied to the parts. A pharmacien of Paris has, for some time past, prepared chlorate of potassa in the same manner.—(*Bull. de Thérap.*, lvii., and *Med. Times*, December, 1859.)

### VIII.—*Phosphorus in Paralysis of the Muscles of the Eye.* By M. TAVIGNOT.

M. Tavignot, in localized paralysis of the muscles of the eye, employs with success the following liniment: Walnut-oil, 3xxv; naphtha, 3xiij; phosphorus, gr. iij. Frictions are performed in the evening by means of a piece of flannel; this remaining also fastened around the forehead all night.

M. Travignot also administers the following emulsion internally: Oil of almonds, 3ijss; phosphorus, gr. jss; gum-sirup, 3xxijss; powdered gum, 3ss. To be well shaken when administered, the dose being at first one, and then two and three teaspoonfuls per diem.—(*Ibid.*)

### IX.—*Collyrium in the Ophthalmia of New-born Infants.* By M. FOUCHER.

M. Foucher employs the following collyrium: Glycerin, 3viij; nitrate of silver, gr. jss ad gr. iij. The eye is first washed by injecting a very weak solution of chloride of sodium, and then a drop of the above collyrium is deposited on the internal surface of the eyelids by means of a small camel's-hair pencil.—(*Ibid.*)

### X.—*Injection of Tincture of Aloes in Gleet.* By M. GAMBERINI.

M. Gamberini, of Bologna, states that in some cases in which other injections have failed, he has derived great advantage from injecting tincture of aloes, fourteen parts diluted with one hundred and twenty parts of water.—(*Ibid.*)

XI.—*On the Treatment of Blennorrhagia by Vinum Colchici and Tincture of Opium.* By DR. EISENMANN, of Würzburg.

Dr. Eisenmann states that he once had occasion to prescribe a combination of vinum colchici and tincture of opium for an officer affected with rheumatic conjunctivitis, and a few days afterwards he was informed that the medicine had cured not only the ophthalmia, but also a blennorrhagia, of which no mention had been previously made. He was surprised at this result; but he resolved to profit by it and to try the same treatment in other cases. He therefore prescribed the medicine for a girl affected with blennorrhagia, and was again surprised that a permanent cure was effected in a few days. Nothing was ordered externally, except frequent applications of tepid water. Subsequently, several cases of blennorrhagia in the male presented themselves, and were treated in the same manner. The dose employed was eighteen to twenty drops, three times a day, of a mixture consisting of twelve grammes of vinum colchici with two grammes of tincture of opium. Milk was ordered as the principal article of food, and absolute rest was enjoined. All the cases of blennorrhagia thus treated were cured, without exception, in a few days, especially when the treatment could be adopted at the commencement of the affection, and none resisted longer than a week.

The observations of Dr. Eisenmann have been confirmed by those of M. Collin, of Dresden, who treated ten cases of blennorrhagia with the greatest success by the mixture of vinum colchici and laudanum. The patients did not recover so rapidly as those treated by Dr. Eisenmann; but the latter physician attributes the difference to the probable inferiority of the drugs employed, and to the fact that the patients did not consult a medical man at a sufficiently early period.—(*Bullet. Génér. de Thérapeutique*, and *Brit. and For. Med.-Chir. Review*, January, 1860.)

XII.—*On Chinese Medicines.* By DR. SCWARTZ.

The Chinese apothecary prepares roots, barks, leaves, fruits, seeds, resins, oils, alkaline earths, metals, crystals, animal bodies and their several parts, especially their secretions and excretions, into infusions, decoctions, powders, pills, extracts, secret preparations, salves, plasters, etc. The mixtures, decoctions of plants, solutions of salts, etc., acquire, by the addition of brown sugar and of mucous and gelatinous substances, a pretty uniform appearance and a similar taste. The soluble substances are often prepared before the patient in an enormous quantity of infusion of tea, and he drinks the medicine and the vehicle in the hot state. The powders are sold in small porcelain and stone jugs, and the stoppers, being unscrewed, have on their inner surface a little bone spoon, with which the medicine is drawn out. The pills, which are uniform and very beautifully rolled, and often gilded, are packed in air-tight, white, transparent wax globes, containing one or two doses. The plasters and salves, usually spread upon red cloth, have a variously-colored paper envelope written over with explanations and praises of the remedy.

The Chinese divide their remedies into two great classes, namely: those which produce fat, and aphrodisiacs. A large paunch is considered a great title to admiration, and the devotion of this extraordinary people to the fair sex is well known.—(*Zeitschrift der Gesellschaft der Aertze zu Wien*, September 19, 1860, and *British and Foreign Med.-Chir. Review.*)

XIII.—*Powder for Chronic Coryza.* By M. MONNERAT.

M. Monnerat has, for some time, observed that subnitrate of bismuth might be employed with advantage as a local application in the acute stage of coryza. When the affection has become chronic, it no longer yields to the bismuth salt employed alone. In such cases, Dr. Sobrier states that he has found it useful to add the iodide of sulphur. His formula consists of four grammes of subnitrate of bismuth, eight grammes of powdered liquorice, and thirty centigrammes of iodide of sulphur. He prescribes ten or twelve pinches, or more, during the day, according to the results obtained.—(*Bullet. Gén. de Thérap.*, and *British and Foreign Med.-Chir. Review*, January, 1860.)

XIV.—*On the Use of Garlic and Lemon-juice in Cases of Membranous Angina.* By DR. CAZIN.

Dr. Cazin, of Boulogne-sur-Mer, gives an instance of membranous angina (diphtheria) successfully treated by garlic. The patient was a young lady, aged fifteen, who presented an extreme degree of swelling of the throat; the whole of the pharynx was covered with a membranous exudation, which was only partially diminished by caustic; the nasal fossæ were affected and poured out an abundant secretion, a symptom always considered unfavorable in the Boulogne epidemic; the pulse was small; the anxiety very great; and the strength was almost exhausted. The citro-alliaceous wash, and draughts of the same kind, were immediately prescribed. From the first day of this treatment there was a marked improvement; the pulse became stronger; the local symptoms were relieved; the membranous exudations were gradually detached; the swelling of the neck was reduced; the throat and nasal fossæ became free, and convalescence was established at the end of five days.—(*Ibid.*)

XV.—*Tannate of Bismuth in Diarrhœa.* By M. CAP.

M. Cap, who has introduced the therapeutic use of glycerin, knowing that tannic acid cures diarrhœa, and that subnitrate and oxide of bismuth have the same effect, thought that it would be advantageous to associate both substances, and has concocted a tannate of bismuth, which he made the subject of a paper at the Academy of Medicine of Paris. Some experiments made by M. Demarquay and M. Trousseau tend to prove that this salt possesses anti-diarrhœic properties. But what sort of diarrhœa does it cure? Because there are very many pathological states of which looseness of the bowels may be a consequence, and the same medicine does not affect them all with equal advantage. In the actual state, the subnitrate of bismuth, though it may be considered as slightly astringent by many therapeutists, especially by M. Pidoux, is far from answering all the indications of tannic acid. They are two agents equally good in their respective spheres of action. By combining them, do we join their advantages or diminish the particular value of each? This is a very legitimate inquiry, and one which M. Bussy has touched on with much good sense. M. Chatin even said, very seriously, that perhaps the merit of the new preparation consisted in its being decomposed in the intestines, and in depositing there separately tannic acid and bismuth. For the present we ask no more, as M. Bouley wishes, than a fair trial, and our remarks are only suggested by a sentiment of general antagonism to the tendencies to polypharmacy which appear to be increasing every day.—(*Gazette Hebdomadaire*, December 2, 1859.)

XVI.—*On the Use of Solanaceæ in Tenesmus.* By M. ANSALONI.

M. Ansaloni reports, in his inaugural dissertation, the favorable results which M. F. Leclerc, of Tours, has obtained from the combined use of belladonna or stramonium, and calomel, in dysentery. Fifty grammes of the extract of belladonna or stramonium are applied in the shape of a large plaster over the pubic region, the two extracts being sometimes used alternately, and one centigramme of calomel is administered for several days, mornings and evenings. The tenesmus and all the other symptoms of dysentery are said to diminish quickly under this treatment. M. Leclerc did not observe any symptoms of poisoning from these applications.—(*Bull. de Thérap.*, lvii. p. 97, 1859.)

XVII.—*Selinum Palustre in Epilepsy.* By DR. TH. HERPIN.

Dr. Th. Herpin communicates the history of four cases of idiopathic, and partly of inherited, epilepsy, in which he effected a complete cure by using, for several months, the powder of selinum palustre. He administered one to four ounces during the week, divided in twenty-four doses, of which three to four were taken daily. Distinct symptoms of the physiological effect of the remedy were not observed.

According to M. Fagod, (*Bouchardat's Annuaire de Thérap.* pour 1859, p. 63,) the root and the herb of the *peucedanum austriacum* are still more efficacious than the selinum palustre. He administered two grammes of the powder three times daily.—(*Bull. de Thérap.*, lvi. p. 403, 1859.)

XVIII.—*Chelidonium Majus in Prurigo.* By M. GRAND-CLEMENT.

The author has used the freshly-expressed juice of chelidonium majus with excellent results in prurient eczema, burns produced from nettles, and other affections of the skin accompanied by prurigo. The juice may be mixed with equal parts of glycerin, and can thus be kept for the time when the plant cannot be obtained in the fresh state.—(*Bull. de Thérap.*, lvi. p. 336, 1859.)

XIX.—*On the Oil of Aleurites Tribola.* By M. O'RORKE.

The author praises the purely cathartic property of this oil, which does not produce nausea, as the oils of other euphorbiaceæ often do. It is nearly as mild in its action as the oleum ricini, but is better to take, because it is more fluid, and does not possess either taste or smell. It is amber colored, insoluble in alcohol, drying, and easily saponified with alkalies. The author classifies the oils of the euphorbiaceæ according to the manner in which they act, and the dose required to produce effect, as follows:—1. Oils causing vomiting and purging: croton oil, one to two drops; oil of jatropha carcas, eight to twelve drops; oil of euphorbia lathyris, one to two grammes; oil of anda gomesii, two to three grammes; oil of hura crepitans, five to ten grammes; castor-oil, at least thirty to sixty grammes. 2. Oil causing merely purging: oil of aleurites tribola, thirty to sixty grammes. 3. Oil producing no effect: omphalæa triandra Lindl. 4. Oils the effect of which is unknown: elæococca verrucosa and stillingia sebifera.—(*Bouchardat's Annuaire*, 1859, p. 117.)

XX.—*Iodide of Ammonium in the Treatment of Constitutional Syphilis.* By DR. GAMBERINI.

This remedy has been employed in England, particularly by Dr. Richardson, in the Royal Infirmary of London, in the form of ointment, and, internally, in a dose of one to five grains in scrofula, rheumatism, syphilis—in short, in all the cases where iodide of potassium is generally used. Dr. Gamberini has applied it on a greater scale to the treatment of syphilitic diseases. In the fourteen cases which he subjected to the experiment, his expectation was answered by success.

According to the author, the iodide of ammonium, called also hydriodate of ammonia, is indicated in all cases where iodide of potassium or sodium are employed. The dose of the medicine is two to sixteen grains daily.

Intolerance of it is experienced only in exceptional cases, and manifests itself by a burning sensation in the throat, and a sense of heat in the stomach, which cease rapidly when the use of the medicine is suspended for a day or two.

The external use of this iodide, three grains to an ounce of olive oil, aids in the removal of the nocturnal syphilitic pains of the muscles and joints. M. Gamberini finds the iodide of ammonium preferable to that of potassium or sodium:—

1. Because, while it produces the same therapeutic effect as the other alkaline iodides, it has the advantage of acting more promptly than they. 2. Because it requires large doses of iodide of potassium or sodium to obtain the results which are procured by a very small dose of iodide of ammonium.—(*Union Medicale*, and *Journal de Pharmacie et de Chimie*, November, 1859.)

---

### TOXICOLOGY AND LEGAL MEDICINE.

I.—*A Case of Emphysema of the Lungs in a Still-born Child; with Observations on the Hydrostatic Test.* By PROF. HECKER.

Professor Hecker reports a rare and interesting case in which he found the lungs of a still-born child in an emphysematous condition. The post-mortem examination was made six hours after the birth of the child; not the least sign of putrefaction could be discovered, so that the presence of air in the lungs could not be ascribed to this cause. On opening the thorax the unusual appearance of the lungs attracted immediate attention; instead of having to be searched for at the bottom of the thorax, they filled out that cavity to its greatest extent, and the left lung covered the pericardium in a manner only observed in cases where respiration has been completely established; their color was not reddish brown as that of fœtal lungs, but much lighter, grayish red; their touch was spongy. Both lungs swam completely, and on being put to the bottom of the vessel, readily came to the surface again. They were well filled with blood, so that on cutting into the parenchyma foamy blood escaped; in many places on their surface, but particularly in their margins, the unmistakable signs of emphysema existed; it resembled that produced when in a case of asphyxia the lungs are incautiously inflated by means of an elastic catheter introduced into the windpipe, and the child has perished soon after the operation; large vesicles filled with air alternated with perfectly white spots. The windpipe, down to the smallest bronchi, was empty, and its mucous membrane somewhat reddened. The heart contained much dark, coagulated blood; its vessels, as well as the fœtal communications, were normal. As an isolated putrefaction of the lungs which might have caused a development of air could not be assumed, Professor Hecker ascribed the appearance of the organs to intense respiratory efforts made in the uterus. The liquor amnii had been very early discharged, and the child was thus enabled to breathe for seventeen hours; furthermore, frequent examinations with half the hand had been made during labor, so that air was repeatedly admitted into the uterus. As motive for respiration no other circumstance can be adduced, as the close embrace of the child by the uterus after the discharge of the liquor amnii; another cause which might have given rise to disturbance in the placental circulation could not be detected.

This case is of particular importance in a medico-legal point of view, as the lungs of this child, which had not breathed at all after birth, were capable of swimming. It invalidates the statement of Casper, that there exists not a single well-observed and indubitable case where emphysema was spontaneously developed in the fœtal lungs, and that it was therefore not admissible in medico-legal practice to ascribe the floating of the lungs of new-born infants to this cause.

As counterpart to the case reported above, Professor Hecker communicates another, which is without parallel. In a child which after birth had breathed and screamed loudly, and which died six hours afterwards, the lungs contained no trace of air and sank completely in water. Their color was that of fœtal lungs, and even a most scrutinizing examination could not discover dilated air-cells in them.—(*Virchow's Archiv für Pathologische Anatomie und Physiologie*, xvi. 5, 6, 1859.)

### II.—*Fallacy of Sources of Arsenic in Dead Bodies.* By DR. LOIS.

Dr. Lois records the fact of his having made examinations of various specimens of brass. He discovered arsenic in ten specimens of brass, and in some specimens in large quantities. He considers this point as of importance to the medical jurist, inasmuch as various brazen ornaments—such as medals and rosaries, which are laid so frequently in coffins—may, by undergoing change in the presence of the products of decomposition, supply to the remains the poison which a chemist may be instructed to search for in the exhumations of persons who have been thought to die from poison.—(*Oesterreichische Zeitschrift für Prak. Heilkunde*, No. xlix., 1859, and *British and Foreign Med.-Chir. Review*.)

### III.—*Death from Chloroform.*

We regret to have to announce another fatal accident during the administration of chloroform for the purpose of producing anæsthesia in a surgical operation. The unhappy patient was Dr. Renwick, of Altoa, a member of our own profession, and but twenty-seven years of age. His disease was in-growing of the great toe-nail, which it was proposed to remedy by evulsion. Dr. Renwick had previously inhaled chloroform without any bad result, hence, perhaps, a false sense of security. The circumstances are thus related:—

A little of the chloroform was poured upon a towel, and he held it to his mouth with his own hands. After awhile, as it did not seem to be taking any effect, he asked for some more, which Dr. Duncanson at first declined to give; but after awhile, finding that no effect was being produced, some more was applied. Observing that he was endeavoring to hasten its effect by strained inspirations, he was asked to breathe naturally, which he did. As it still, however, seemed to be having no effect, another small quantity, at his own request, was applied to the towel, which after a time produced insensibility; and his pulse having been found full and regular the operation, which did not occupy more than a minute or two, was successfully performed. He still remained under the influence of the anæsthetic, but his breathing was regular, and all was considered right. Some cold water was then thrown on his face to arouse him; but this not having the desired effect other measures were resorted to, but with a like unfortunate result; and when, after a few minutes, his breathing became less frequent and more labored, and the appearance of his countenance began to change, and his pulse had become nearly imperceptible, serious alarm was felt. Artificial respiration by the modern method was resorted to, and in this manner breathing was kept up for nearly half an hour; but, melancholy to relate, his spirit had passed away.

We are unwilling to add any observations which can give pain, but it is certainly to be lamented that Snow's inhaler, or some other efficient apparatus was not employed. There is some reason to believe that Dr. Renwick was the subject of cardiac disease.

The *North British Mail* mentions that some time ago a gentleman died under the influence of chloroform at Girvan, while undergoing a similar operation.—(*Lancet*, January 7, 1860.)

### IV.—*Poisoning by Cyanide of Potassium.*

Dr. A. Schauenstein, in a communication on poisoning by cyanide of potassium, gives an account of five cases of death by the cyanide. The author, who is a judicial chemist, comments upon the great increase of deaths by suicide through the agency of this poison. Thus in Vienna, from 1851 to 1856 only two poisonings were noted, one of which was doubtful; while from August, 1857, to December, 1858, no less than five cases came under the personal observation of the author. In proportion to this increase of deaths from the cyanide there was a corresponding decrease of deaths from arsenic.

Dr. Schauenstein relates at length three of the cases observed, and in brief the pathology of the two others. In all cases the death seems to have been sudden. In one case, in a young girl, strong tetanic spasms came on directly after the poison had been taken, and death took place in less than an hour. In the second case, occurring in a young man, death took place almost instantly, and with no striking symptoms. The third case was similar; no note of the symptoms in the remaining two cases is given, but Dr. Schauenstein observes, that in several of the cases death took place suddenly, as in apoplexy.

In all the cases a post-mortem examination was conducted, but the appearances observed are considered by the author as offering nothing very characteristic. They were:—

(*a*) The brain contained more or less blood.

(*b*) The blood in the cavities of the heart dark, and of thick consistency.

(*c*) The condition of the stomach varies. In one case the mucous surface presented no particular coloring. In the case where life was prolonged nearly an hour the mucous membrane was slightly red, but offered no other extraordinary appearance. In another case, the death being very sudden, the mucous membrane was of a dark-red color, swollen, and in places covered with numerous bloody points; the contents of the stomach were also of blood-red color; the two remaining cases of the five presented similar appearances in a less degree.

(*d*) The smell of prussic acid in the stomach was very evident in four of the cases. But in one case, on account of the quantity of undigested food in the stomach, the smell remained hidden entirely.

(*e*) The reaction of the contents of the stomach was strongly alkaline, and in every case chemical search proved without doubt the presence of prussic acid; but formic acid was also constantly found, showing that prussic acid in the stomach is transformed into formic acid in many cases.

This latter fact, one of great interest, was originally pointed out by Dr. Schauenstein in the *Wochenbl. der Zeitschrift der K. K. Gesellschaft der Aerzte*, No. 3, 1857.

Dr. Schauenstein, in commenting on the cases, opines that there are no true and distinguishing pathological indications by which the effects of the poison can be safely pronounced.

He further observes that the chemical detection may become equally difficult in instances where, from the body having been dead several days, or having undergone a rapid decomposition, the poison has been decomposed.—(*Zeitschrift der K. K. Gesellschaft der Aerzte zu Wien*, No. 1, 1859, and *British and Foreign Med.-Chir. Review.*)

